# Evaluating the Discriminatory Ability of the Sickle Cell Data Collection Program’s Administrative Claims Case Definition in Identifying Adults With Sickle Cell Disease: Validation Study

**DOI:** 10.2196/42816

**Published:** 2023-06-28

**Authors:** Ashima Singh, Marci K Sontag, Mei Zhou, Mahua Dasgupta, Tessa Crume, Morgan McLemore, Najibah Galadanci, Eldrida Randall, Nicole Steiner, Amanda M Brandow, Kathryn Koch, Joshua J Field, Kathryn Hassell, Angela B Snyder, Julie Kanter

**Affiliations:** 1 Department of Pediatrics Medical College of Wisconsin Milwaukee, WI United States; 2 Center for Public Health Innovation CI International Littleton, CO United States; 3 Georgia Health Policy Center Andrew Young School of Policy Studies Georgia State University Atlanta, GA United States; 4 Department of Epidemiology Colorado School of Public Health University of Colorado Anschutz Medical Center Aurora, CO United States; 5 Department of Hematology and Oncology Winship Cancer Institute Emory School of Medicine Atlanta, GA United States; 6 Department of Medicine University of Alabama Birmingham Birmingham, AL United States; 7 Department of Medicine Medical College of Wisconsin Milwaukee, WI United States; 8 Versiti Blood Center of Wisconsin Milwaukee, WI United States; 9 University of Colorado Aurora, CO United States

**Keywords:** surveillance using administrative data, rare conditions, sickle cell disease, disease, surveillance, genetic, prevention, data, adults, epidemiology, utilization

## Abstract

**Background:**

Sickle cell disease (SCD) was first recognized in 1910 and identified as a genetic condition in 1949. However, there is not a universal clinical registry that can be used currently to estimate its prevalence. The Sickle Cell Data Collection (SCDC) program, funded by the Centers for Disease Control and Prevention, funds state-level grantees to compile data within their states from various sources including administrative claims to identify individuals with SCD. The performance of the SCDC administrative claims case definition has been validated in a pediatric population with SCD, but it has not been tested in adults.

**Objective:**

The objective of our study is to evaluate the discriminatory ability of the SCDC administrative claims case definition to accurately identify adults with SCD using Medicaid insurance claims data.

**Methods:**

Our study used Medicaid claims data in combination with hospital-based medical record data from the Alabama, Georgia, and Wisconsin SCDC programs to identify individuals aged 18 years or older meeting the SCDC administrative claims case definition. In order to validate this definition, our study included only those individuals who were identified in both Medicaid’s and the partnering clinical institution’s records. We used clinical laboratory tests and diagnostic algorithms to determine the true SCD status of this subset of patients. Positive predictive values (PPV) are reported overall and by state under several scenarios.

**Results:**

There were 1219 individuals (354 from Alabama and 865 from Georgia) who were identified through a 5-year time period. The 5-year time period yielded a PPV of 88.4% (91% for data from Alabama and 87% for data from Georgia), when only using data with laboratory-confirmed (gold standard) cases as true positives. With a narrower time period (3-year period) and data from 3 states (Alabama, Georgia, and Wisconsin), a total of 1432 individuals from these states were included in our study. The overall 3-year PPV was 89.4% (92%, 93%, and 81% for data from Alabama, Georgia, and Wisconsin, respectively) when only considering laboratory-confirmed cases as true cases.

**Conclusions:**

Adults identified as having SCD from administrative claims data based on the SCDC case definition have a high probability of truly having the disease, especially if those hospitals have active SCD programs. Administrative claims are thus a valuable data source to identify adults with SCD in a state and understand their epidemiology and health care service usage.

## Introduction

Sickle cell disease (SCD) is a rare genetic condition, which has consistently suffered from disparity in terms of research funding, availability of registries, and surveillance programs [1]. Recently, the National Academies of Sciences, Engineering, and Medicine (NASEM) published a strategic plan and blueprint for action addressing SCD [2]. One of the recommendations of the NASEM report includes establishing a nationwide surveillance program for SCD in addition to a longitudinal clinical registry. A clinical registry is an organized system of data collection using observational study methods to evaluate specified outcomes for a population defined by a particular disease or condition (in this case, SCD). In most cases, a registry requires patient consent to participate, which may limit the inclusion of all individuals with the specific condition. In contrast, surveillance data are defined as the ongoing, systematic collection, analysis, and interpretation of health-related data needed for the planning, implementation, and evaluation of public health practice [2]. While a registry can inform on clinically specific information including disease trajectory, a surveillance program provides a more population-based assessment and is often more inclusive of the entire population. The Centers for Disease Control and Prevention has invested in establishing the Sickle Cell Data Collection (SCDC) [3] program to establish a population-based surveillance program in multiple US states using a combination of data sources. A population-based surveillance program for SCD both helps to reveal the distribution of individuals living with SCD and inform stakeholders about the health care usage patterns to improve outcomes. Understanding the patterns of care for people with SCD can increasingly ensure sufficient staffing, and expertise is available to treat the patient population.

Administrative claims data are one of the data sources used in the SCDC population-based surveillance program. Currently, all states participating in the SCDC program include or plan to include data from different administrative sources as they establish their state-specific programs in addition to data from newborn screening programs (NBSs), SCD center–specific databases, electronic health records data warehouses, and vital health records. In contrast to data from NBS programs or clinical databases, claims data are only based on codes of the International Classification of Diseases, Ninth and Tenth Revisions (ICD-9 and -10, respectively), and not clinically verified data. Despite this limitation, claims data are a valuable source of information since they include comprehensive information on health care service usage for their beneficiaries beyond specific hospitals and clinics. This is specifically important for the SCDC surveillance efforts, which aim to include all individuals living with SCD in participating states. Prior work has validated the definition of ≥3 SCD-coded encounters within 5 years to identify a pediatric patient cohort at a large urban children’s hospital [4]. This definition, however, has not been tested in the adult population. As the SCDC program expands to multiple states, the generalizability and validity of the SCDC administrative case definition needs to be determined. Also, some of the recently participating states have limited years of available data. The objective of our study is for 3 of the SCDC states to evaluate the discriminatory ability of the SCDC administrative case definition to identify adults with SCD (≥18 years of age) who are Medicaid beneficiaries, using Medicaid insurance claims data from their state, considering confirmatory laboratory assessment of SCD as the gold standard. We hypothesize that the SCDC case definition applied to Medicaid claims data will have a high positive predictive value (PPV) for identifying SCD cases among people with SCD aged ≥18 years, but that it will be lower than that observed among pediatric cases.

## Methods

### Administrative Claims Data Sources to Identify Adults With SCD

Our study includes Medicaid claims data from the Alabama, Georgia, and Wisconsin SCDC programs.

### Inclusion Criteria

Adult individuals (aged 18 years or older at the beginning of the study period) with 3 or more claims with an SCD ICD code (ICD-9: 282.41, 282.42, and 282.6*; ICD-10: D57.0*, D57.1, D57.2*, D57.4*, and D57.8*) in their state-specific Medicaid data were eligible for inclusion. In addition, eligible individuals also needed evidence of at least 1 visit (with any diagnosis) at the specific clinical partner institutions. Our 5-year time frame analysis included data from Alabama and Georgia. Eligible individuals identified within Alabama Medicaid claims data from 2015 to 2019 were cross-referenced with electronic health record data from the University of Alabama at Birmingham’s hospital system. Eligible individuals identified within Georgia’s Medicaid claims data from 2015 to 2019 were included if any claim contained a Grady Memorial Hospital facility code. Our 3-year time frame analysis included data from Alabama, Georgia, and Wisconsin. The inclusion criteria for Alabama and Georgia were identical, as described above, with a restricted time frame of 2015-2017. The Wisconsin data included eligible individuals identified within Medicaid claims data from 2018 to 2020 who were linked and matched with electronic health record data from Froedtert Hospital.

### Ethics Approval

All states providing data had approvals or exemption to conduct the project. Specifically, the University of Alabama Institutional Review Board (IRB) approved the project as Non-human subjects research (IRB-300004733), the Georgia State University's IRB approved the state's SCDC program under a public health exemption (protocol #H11142), and the Medical College of Wisconsin IRB granted an exemption for the study in accordance with 45 CFR 46.104(d)(4) (PRO00043293).

### Validation Data

SCD centers from the University of Alabama at Birmingham, Grady Memorial Hospital, and Froedtert Hospital contributed their patient-level databases, which included people with “confirmed” SCD. Hematologists at each site also reviewed the laboratory reports of all the individuals identified at their institutions as part of this study. An individual had confirmatory evidence of SCD if he or she had a confirmed Clinical Laboratory Improvement Amendments of 1988–certified laboratory result from a hemoglobin electrophoresis or high-performance liquid chromatography test in their medical records. All patients with confirmed SCD had these laboratory tests in their medical records (even if they were not active patients within the SCD center). For individuals without evidence of confirmatory laboratory testing, a clinical algorithm was used to evaluate nondiagnostic laboratory data available in the electronic medical record system to classify them as being “likely” to have SCD. The clinical algorithm considered individuals “likely” to have SCD if they had at least 15 laboratory values that included a total bilirubin level of >1.1 mg/dL and any one of the following: reticulocyte proportion of >2%, lactate dehydrogenase level of >250 units/L, or hemoglobin level of <11 g/dL. Individuals with a hemoglobin analysis in their medical records, confirming that they did not have SCD (including those with the sickle cell trait), were categorized as “do not have SCD.” Individuals who did not have confirmatory laboratory evidence regarding their SCD status or those who were not classified as likely cases were labeled as “indeterminant” cases, implying that there was not enough evidence in their medical record to make a diagnosis.

**Figure 1:**
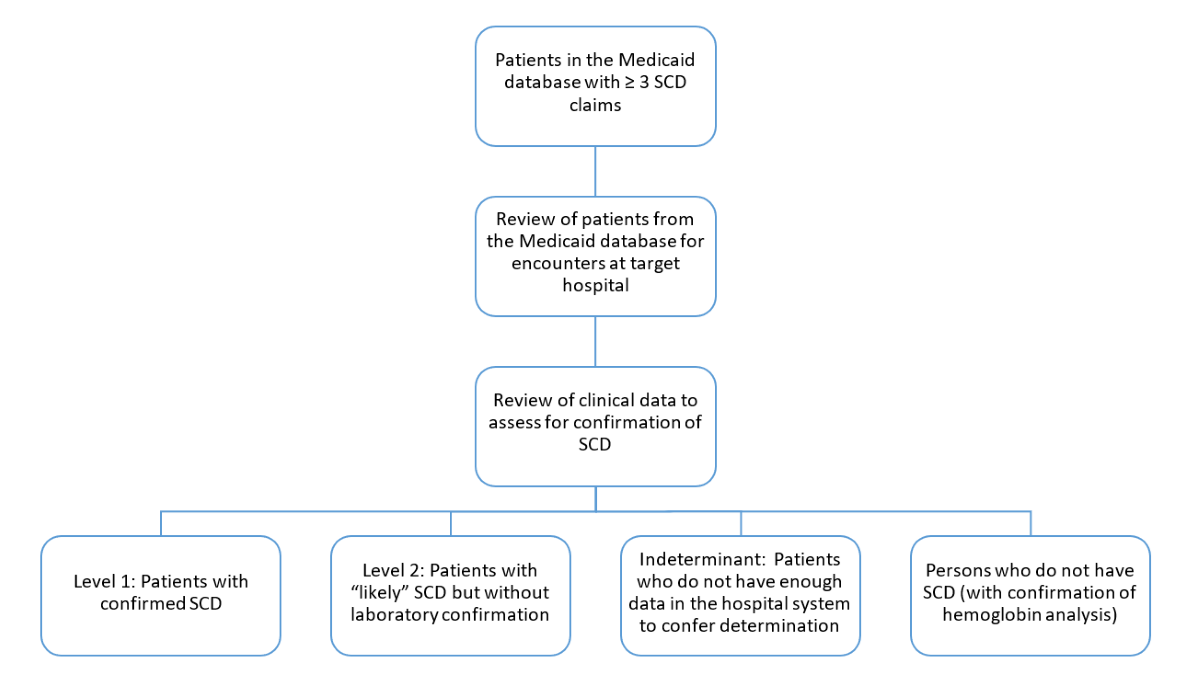
Overview of study methodology. SCD: sickle cell disease.

The stepwise method of identification of the eligible population and the classification of cases into various categories is illustrated in Figure 1.

### Analysis

Summary statistics were calculated to describe the demographic characteristics of age, sex, and race and ethnicity based on information available in each state’s Medicaid data. Analysis was conducted for 2 time periods based on available data: 5 years (Alabama and Georgia) and 3 years (Alabama, Georgia, and Wisconsin). We determined the PPV, overall and by state, under various scenarios for the respective time frames. In the first scenario, true positives (TPs) included individuals “confirmed” to have SCD. In the second scenario, TPs also included individuals “confirmed” and “likely” to have SCD. The PPV was calculated using the TP as the numerator and the total number of SCD cases identified per the definition as the denominator (TPs+false positives [FPs]). As an alternate strategy, we also calculated a best-case PPV excluding individuals with an “indeterminant” SCD status for both the scenarios described above. This was done because “indeterminant” cases did not have sufficient information to be either classified as TP or FP. Exact binomial 95% CIs were reported for all proportions. For the group of patients recognized as FP cases based on confirmatory evidence of not having SCD in their clinical data, we provide a count of those with the sickle cell trait.

## Results

### Overview

There were a total of 3425 (Alabama, n=935; Georgia, n=2490) and 3365 (Alabama, n=803; Georgia, n=2055; Wisconsin, n=507) individuals who were aged ≥18 years and had at least ≥3 Medicaid SCD claims in the 5-year and 3-year time frame, respectively. A total of 1219 unique individuals (354 from Alabama and 865 from Georgia) in the 5-year time period and 1432 (328 from Alabama, 684 from Georgia, and 420 from Wisconsin) in the 3-year time period had at least 3 Medicaid SCD claims and an encounter with the respective partner clinical institution. Importantly, not all of those individuals with SCD claims were seen by the sickle cell center at their respective institutions (ie, they only had to be seen within the hospital system). The demographic characteristics and the total number of SCD-related Medicaid encounters are described in Table 1.

**Table 1:** Demographic characteristics of individuals included in the study.

Characteristics			Values															
			Total					Alabama				Georgia				Wisconsin		
			5-year time frame (n=1219)		3-year time frame (n=1432)		5-year time frame (n=354)		3-year time frame (328)		5-year time frame (n=865)			3-year time frame (n=684)	5-year time frame			3-year time frame (n=420)
Age (years), mean (SD)			30.1 (10.7)		32.1 (11.3)		29.0 (9.3)		28.8 (9.4)		30.5 (11.2)			30.03 (10.8)	N/A^a^			38.0 (11.4)
**Age group (years), n (%)**																		
	18-29	735 (60)		731 (51)		237 (67)			221 (67)	489 (57)			397 (58)		N/A		113 (27)	
	30-39	299 (25)		396 (28)		76 (21)			68 (21)	223 (26)			175 (26)		N/A		153 (36)	
	40-49	119 (10)		184 (13)		26 (7)			24 (7)	93 (11)			70 (10)		N/A		90 (21)	
	50-59	49 (4)		88 (6)		12 (3)			12 (4)	37 (4)			27 (4)		N/A		49 (12)	
	≥60	26 (2)		36 (2)		3 (1)			3 (1)	23 (3)			15 (2)		N/A		18 (4)	
Sex (female), n (%)			743 (61)		847 (59)		224 (63)		209 (64)		519 (60)			396 (58)	N/A			242 (57)
SCD^b^ encounters per patient during the study period, mean (SD)			64.5 (80.5)		42.3 (51.4)		47.8 (33.5)		31.4 (20.2)		71.4 (92.3)			51.1 (61.7)	N/A			36.5 (47.2)

^a^N/A: not applicable.

^b^SCD: sickle cell disease.

### PPV Based on Data With a 5-Year Time Period

Table 2 shows the demographic characteristics of individuals in each validation category by state. The mean age of individuals with confirmed SCD from Alabama and Georgia was 27.9 (SD 8.6) years and 29.8 (SD 10.7) years, respectively, with the majority of them being in the 18-29–year age group. Overall, 69% of individuals with confirmed SCD across Alabama and Georgia had Hemoglobin SS/Sβ0 type of disease. PPV values by state are shown in Table 3. The overall PPV was 88% when considering only those with a confirmatory clinical laboratory test indicating SCD as TPs. For the scenario that included both likely and confirmed cases as TP, the PPV was slightly higher (89%).

Overall, across Alabama and Georgia, there were 58 cases (14 from Alabama and 44 from Georgia) that were FP (cases with confirmatory evidence of not having SCD). Of them, 36% (5 from Alabama and 16 from Georgia) had the sickle cell trait. There were 73 cases that were indeterminate (8 from Alabama and 65 from Georgia). When excluding the indeterminate cases from the denominator (data not shown in tables), the PPVs for the 2 scenarios of TPs were 94% and 95%, respectively. The PPVs (when excluding indeterminate cases from the denominator; data not shown in tables) remained at >83% for both the TP scenarios irrespective of age group and state.

**Table 2:** Demographic characteristics of individuals with confirmed sickle cell disease (SCD), likely SCD, those not having SCD, and those with an indeterminate status identified by the clinical validation process by state over a 5-year period.

		Alabama^a^ (n=354)					Georgia^b^ (n=865)			
		Confirmed SCD (n=323)	Likely SCD (n=9)	Confirmed as not having SCD (n=14)	Indeterminant status (n=8)	Confirmed SCD (n=755)		Likely SCD (n=1)	Confirmed as not having SCD (n=44)	Indeterminant status (n=65)
Age (years), mean (SD)		27.9 (8.6)	32.9 (4.8)	27.3 (5.6)	39.1 (11.7)	29.8 (10.7)		—^c^	30.3 (11.1)	38.7 (13.3)
**Age groups (years), n (%)**										
	18-29	224 (69)	1 (11)	10 (71)	2 (25)	448 (59)		—	26 (59)	15 (23)
	30-39	62 (19)	8 (89)	4 (29)	2 (25)	189 (25)		—	10 (23)	24 (37)
	40-49	23 (7)	—	—	3 (38)	70 (9)		1 (100)	7 (16)	15 (23)
	50-59	11 (3)	—	—	1 (12)	33 (4)		—	—	4 (6)
	≥60	3 (1)	—	—	—	15 (2)		—	1 (2)	7 (11)
Sex (female), n (%)		204 (63)	6 (67)	9 (64)	4 (50)	430 (57)		—	39 (89)	50 (77)
SCD encounters per patient during the study period, mean (SD)		50.1 (33.5)	30 (37)	19.1 (11.3)	17.4 (18.4)	80.2 (96.0)		27 (N/A^d^)	14.0 (15.9)	9.2 (17.6)

^a^SCD types in Alabama: hemoglobin SS/Sβ0, n=205 (63%); hemoglobin SC, n=68 (21%); others, n=50 (15%).

^b^SCD types in Georgia: hemoglobin SS/Sβ0, n=536 (71%); hemoglobin SC, n=176 (23%); others, n=43 (6%).

^c^Not available.

^d^N/A: not applicable.

**Table 3:** The positive predictive value (PPV) of the Sickle Cell Data Collection (SCDC) case definition by state over a 5- and 3-year period.

	5-year time period			3-year time period	
	PPV including only confirmatory cases as TP^a^ (%), PPV (95% CI)	PPV including confirmatory and likely cases as TP (%), PPV (95% CI)	PPV including only confirmatory cases as TP (%), PPV (95% CI)		PPV including confirmatory and likely cases as TP (%), PPV (95% CI)
Alabama	91 (87-94)	94 (91-96)	92 (89-95)		95 (92-97)
Georgia	87 (85-90)	87 (85-90)	93 (91-95)		93 (91-95)
Wisconsin	N/A^b^	N/A	81 (77-85)		82 (78-86)

^a^TP: true positive.

^b^N/A: not applicable.

### PPV Based on Data With a 3-Year Time Period

Table 4 shows the demographic characteristics of individuals in each validation category by state. Similar to the 5-year time frame, the mean age of individuals confirmed with SCD was 28.0 (SD 9.5) years and 29.6 (SD 10.5) years, respectively, with the majority being in the 18-29–year age group. The individuals confirmed with SCD from the Wisconsin records had a higher mean age (36.7, SD 10.5 years), with 30% and 38% of them being in the 18-29–year and 30-39–year age group, respectively. The PPVs for all scenarios by state are shown in Table 3. The overall PPV was 89% when considering only those with a confirmatory clinical laboratory test indicating SCD as TP. For the scenario that included both likely and confirmed cases as TP, the PPV was slightly higher (91%).

Overall, across Alabama, Georgia, and Wisconsin, there were 60 cases (11 from Alabama, 21 from Georgia, and 28 from Wisconsin) that were FP (cases with confirmatory evidence of not having SCD), of whom 50% (5 from Alabama, 9 from Georgia, and 16 from Wisconsin) had the sickle cell trait. There were 79 (6 from Alabama, 25 from Georgia, and 48 from Wisconsin) indeterminate cases. When excluding the indeterminate cases from the denominator, the PPVs for the 2 scenarios of TPs were 95% and 96%, respectively. The PPVs (when excluding indeterminate cases from the denominator; data not shown in tables) remained at >83% for both the TP scenarios irrespective of age group and state.

**Table 4:** Demographic characteristics of individuals with confirmed sickle cell disease (SCD), likely SCD, those not having SCD, and those with an indeterminate status identified by the clinical validation process by state over a 3-year period.

		Alabama^a^ (n=328)					Georgia^b^ (n=684)					Wisconsin^c^ (n=420)			
		Confirmed SCD (n=302)	Likely SCD (n=8)	Confirmed as not having SCD (n=12)	Indeterminant status (n=6)	Confirmed SCD (n=638)		Likely SCD (n=0)	Confirmed as not having SCD (n=21)	Indeterminant status (n=25)	Confirmed SCD (n=340)		Likely SCD (n=4)	Confirmed as not having SCD (n=28)	Indeterminant status (n=48)
Age (years), mean (SD)		28.0 (9.5)	32.9 (5.1)	28.58 (6.3)	36.8 (12.2)	29.6 (10.5)		N/A^d^	31.1 (9.6)	39.2 (14.5)	36.7(10.5)		43.8 (11.5)	37.4(8.6)	46.4 (14.9)
**Age groups (years), n (%)**															
	18-29	210 (70)	1 (12)	8 (67)	3 (50)	380 (60)		N/A	10 (48)	7 (28)	101 (30)		0 (0)	5 (18)	7 (14)
	30-39	55 (18)	7 (88)	4 (33)	2 (33)	160 (25)		N/A	6 (29)	9 (36)	129 (38)		1 (25)	12 (43)	9 (19)
	40-49	23 (8)	—^e^	—	1 (17)	63 (10)		N/A	5 (24)	2 (8)	64 (19)		2 (50)	9 (32)	15 (31)
	50-59	11 (4)	—	—	1 (17)	23 (4)		N/A	—	4 (16)	37 (11)		1 (25)	2 (7)	8 (17)
	≥60	3 (1)	—	—	—	12 (2)		N/A	—	3 (12)	9 (3)		0 (0)	0 (0)	9 (19)
Sex (female), n (%)		192 (64)	7 (88)	7 (58)	3 (50)	358 (56)		N/A	19 (90)	19 (76)	183 (54)		3 (75)	21 (75)	35 (73)
SCD encounters per patient during the study period, mean (SD)		32.4 (20.2)	22 (17)	15.1 (7.1)	16.2 (13.5)	53.9 (62.7)		N/A	12.6 (14.9)	11.7 (26.3)	40.3 (43.7)		7.5 (2.6)	11.3 (20.7)	26.5 (72.0)

^a^SCD types in Alabama: hemoglobin SS/Sβ0, n=199 (66%); hemoglobin SC, n=57 (19%); others, n=46 (15%).

^b^SCD types in Georgia: hemoglobin SS/Sβ0, n=458 (72%); hemoglobin SC, n=146 (23%); others, n=34 (5%).

^c^SCD types in Wisconsin: hemoglobin SS/Sβ0, n=220 (65%); hemoglobin SC, n=86 (25%); others, n=35 (10%).

^d^N/A: not applicable.

^e^Not available.

## Discussion

### Principal Findings

This study supports the use of a standardized surveillance case definition within administrative claims data, specifically using Medicaid claims (≥3 ICD-9 or -10 codes), across multiple states to identify adults living with SCD. The PPVs achieved through systematic application of the case definitions among adults who receive care at hospitals with an SCD program has resulted in PPVs of ~90%, which is laudable. When excluding the indeterminate cases, the PPV of the SCDC administrative case definition for adults is similar to the PPV of >95% demonstrated in pediatric populations [4,5].

The lower PPV in adults may have resulted from inaccurate coding in Medicaid claims. A sizeable proportion of the FPs identified within Medicaid data included individuals with the sickle cell trait. This represents an opportunity to better educate providers and the general population about the distinction between the 2 conditions. The sickle cell trait is a mostly benign condition and individuals do not experience vaso-occlusive pain episodes. In contrast, individuals with SCD are at risk for significant organ damage as well as acute pain episodes. Thus, research that incorporates individuals with the sickle cell trait into the SCD cohort may lead to substantial underestimation of the disease burden. This emphasizes the need for research specific to SCD based on the combination of information from multiple data sets to minimize the inclusion of individuals with the sickle cell trait. This also supports algorithms developed for other types of data sources such as electronic health record data that exclude individuals with the sickle cell trait when defining those with SCD [6,7]. Specifically, the lower predictive value in Wisconsin, as compared to that in Alabama and Georgia, was mainly driven by the number of indeterminant cases. The indeterminant cases did not have enough information to be classified as having or not having SCD. Interestingly, Wisconsin is also the newest state to join the SCDC program, which may be affecting their number of indeterminate cases (and it may change over time). While better education of providers managing people with SCD may result in improved coding practices, these inaccuracies may also occur with ancillary providers, such as those providing radiology or laboratory testing services, who may not directly interact with the patient. These ancillary providers could contribute to both FPs if they assume that a patient has SCD because he or she was referred by a SCD specialist or had been seen at a hematology clinic, as well as to false negatives since they must often assume a patient’s diagnosis based on conditions inaccurately listed on a problem list. Further, many physicians caring for people with SCD also see individuals with other hematologic conditions. Moreover, there are no codes for “hemoglobinopathy screening” to be used by providers assessing these individuals. Some individuals may be miscategorized as having “SCD” simply because they are undergoing hemoglobinopathy testing, which can be carried forward in the medical record. Fragmentation of care for adult patients with SCD [8,9] may also result in less consistent diagnosis and coding.

As efforts continue to improve access to care for adults with SCD, the SCDC program remains an important source of data to characterize SCD including evaluations of SCD management and acute care use on a population level. Administrative data are paramount to creating the surveillance database used by state SCDC programs. While many sickle cell centers have local patient databases, a single current national registry for SCD does not exist, although efforts are underway through the National Alliance of Sickle Cell Centers [10], the National Institutes of Health’s Sickle Cell Disease Implementation Consortium [11], and the American Society of Hematology’s Research Collaborative [12]. However, adults living with SCD may be excluded from these efforts if they do not receive care at one of the participating comprehensive SCD centers. Thus, it is not possible to include the entire population living with SCD in a registry, and performance metrics including sensitivity become impossible to calculate. While efforts are underway to improve access to care for those with SCD [13], it is vital that we have a surveillance program to better understand the health care usage pattern of those living with SCD to determine where additional SCD centers should be established.

Georgia and California were the first 2 states to develop robust SCD surveillance programs. Current SCD surveillance efforts are expanding as the SCDC program receives additional federal funding to extend to additional states. As new states join the SCDC program, it becomes increasingly important to ensure that epidemiologic studies resulting from these data provide an accurate portrayal of SCD in terms of its morbidity and mortality across all age groups. Notably, PPVs consistently over 80%, even among older age groups, support the use of these data to track survival and other outcomes in SCD over the individuals’ lifespans with reasonable accuracy. These findings add to the data supporting state programs in equitable resource allocation and in determining the best strategies to improve outcomes and quality of life for those living with SCD. Administrative data are especially important in identifying adults who are too old to have been screened for SCD at birth (ie, missed the NBS window) as well as those adults who have little access to SCD centers. Additionally, these findings demonstrate the importance of continued data collection and support for the SCDC program, which will allow for additional refinement of surveillance-based case definitions for SCD.

### Limitations

There are several limitations to this study. First, individuals who do not have SCD are not included in the SCDC program. Thus, we are unable to comment on the number of false negative codes within the data sets. As a result, it is not feasible to assess the specificity of the data definition on a population level. In addition, these studies were conducted at hospitals that treat a large number of individuals living with SCD (and have National Alliance of Sickle Cell Centers–recognized SCD centers). Thus, it is possible that hospitals that. Although we report a conservative estimate of the definition’s PPVs, the performance of the definition might differ at nonacademic community-based hospitals or those in rural settings or both. Finally, there are several indeterminate cases where insufficient laboratory data were available to confirm an SCD diagnosis. This limitation highlights the importance of obtaining baseline data for persons with SCD newly seen at any hospital system. Confirming an SCD diagnosis is clinically important for medical management and should not be assumed based on a previously notated problem in the medical history or a previous administrative code. Instead, providers need to ensure that all individuals who present with a diagnosis of SCD (or having a chief complaint of an SCD-related symptom) have a hemoglobin analysis performed if never previously evaluated.

### Conclusions

Overall, these data validate the use of the administrative case definition identified by the SCDC programs to identify adults with SCD. While there are limitations to the use of this definition, it can be highly valuable for SCD surveillance to improve the understanding of the patterns of health care usage in the SCD population over time.

## Abbreviations

FP: false positive
ICD: International Classification of Diseases
NASEM: National Academies of Sciences, Engineering, and Medicine
NBS: newborn screening program
SCD: sickle cell disease
SCDC: Sickle Cell Data Collection
TP: true positive
